# Incidence of acute diarrheal illness in Chinese communities: a meta-analysis

**DOI:** 10.1186/s12876-018-0839-2

**Published:** 2018-07-13

**Authors:** Pengwei Cui, Jingxin Li, Na Liu, Zhao-jun Duan

**Affiliations:** 1 Suzhou Center for Disease Control and Prevention, Jiangsu Province, Suzhou, China; 2 Department of Viral Diarrhea, National Institute for Viral Disease Control and Prevention, Chinese Centers for Disease Control and Prevention, 155 Changbai Road, Changping District, Beijng, 102206 China

**Keywords:** Acute diarrhea illness, Incidence, Disease burden, China

## Abstract

**Background:**

Acute diarrheal illness (ADI) is an important public health problem worldwide. We estimated the morbidity, distribution, and burden of self-reported ADI in China over the last three decades.

**Methods:**

We used the keywords “diarrhea and morbidity” to identify studies published in Chinese by searching CNKI, WANFANG, Chongqing VIP, and SinoMed. Studies published in English were identified using the keywords “diarrhea, morbidity, and China” to search Pubmed/Medline, Embase, and Cochrane Library Data. All articles published before Dec 31, 2014 were included in the search. Data were extracted and the pooled 2-week incidence rate of ADI was calculated using the fixed-effects or random-effects model according to statistical testing for homogeneity. The incidences of each subgroup (organized by age, location, study period) were also calculated. Publication bias was examined using Begg’s test. Data manipulation and statistical analyses were undertaken using R-2.15.1 software.

**Results:**

We estimated that the pooled 2-week prevalence of ADI in China was 2.04% (95% CI: 1.48–2.79) and that the corresponding incidence rate was 0.53 (95% CI: 0.38–0.73) episodes per person-year. The ADI rate was highest among children aged < 5 years (1.43 episodes per person-year), and it was slightly higher in males than in females (0.58 vs 0.52 episodes per person-year). From 1980 to 2012, there was a significant decrease in the incidence of ADI, from 0.82 to 0.48 episodes per person-year, but the ADI incidence was consistent over the last two decades. Additionally, the incidence of ADI was higher in rural areas and in west China and peaked in the summer months.

**Conclusions:**

The current study indicates that ADI caused a substantial disease burden in China in the last 30 years, especially in rural areas and west China, where sanitation conditions were relatively poor. These findings highlight the importance of further investigation of the specific causes of and effective preventive measures for ADI.

**Electronic supplementary material:**

The online version of this article (10.1186/s12876-018-0839-2) contains supplementary material, which is available to authorized users.

## Background

Acute diarrheal illness (ADI) is a common and important public health problem worldwide [1, 2]. It is one of the leading causes of morbidity and mortality in children younger than 5 years of age (YOA) and is responsible for the deaths of about 577,508 children worldwide every year, predominantly in developing countries [3]. Diarrhea incidence among children under 5 YOA has declined from 3.4 episodes/child year in 1990 to 2.9 episodes/child year in 2010 worldwide [4]. Diarrhea morbidity rates range from 29.9 episodes/100 person-years for adults in the South East Asian region to 88.4 episodes/100 person-years in older children in the Eastern Mediterranean region and have remained unchanged in the last 30 years [5]. China remains one of the countries with the highest mortality rates due to diarrhea, with nearly 9072 deaths among children younger than 5 YOA every year [3]. In developed countries, ADI is rarely fatal and is usually self-limiting, but it causes considerable morbidity and high costs due to consultations, medications, hospitalizations, and absence from work [6–8].

Given the prevalence of ADI in the community, China has established several national diarrhea surveillance systems based in healthcare facilities or laboratories [9, 10], including the National Notifiable Disease Reporting System, the National Foodborne Diseases Surveillance Network, the National Viral Diarrhea Surveillance System, and so on. However, not all ADI cases present to healthcare facilities, and a high proportion of those who do report are not reported to the national surveillance systems [11, 12]. Thus, many episodes of ADI cannot be tracked by routine surveillance systems, leading to underestimates of the ADI burden. Cross-sectional surveys in the community are a better way to estimate the burden of ADI [8, 13, 14].

In China, several national or regional population-based studies on the ADI burden have been conducted as a part of National ADI surveillance systems [15, 16], but these studies reported a 10-fold difference in reported ADI incidences, with figures ranging from 0.13 to 1.16 episodes per person-year [17, 18]. Diarrhea specific incidence rates for older children, adolescents, and adults have not been systematically calculated in China. Therefore, to better understand the ADI incidence in China, we conducted the meta-analysis described herein.

## Methods

### Search strategy

Using the terms “diarrhea” and “morbidity,” as well as “China” if published in English, studies were identified from the following electronic databases: Pubmed/Medline, Embase, Cochrane Library, Chinese National Knowledge Infrastructure, Wanfang Data, Chongqing VIP database for Chinese Technical Periodicals, and China BioMedical Literature Services System (SinoMed). No attempt was made to retrieve unpublished studies. All articles published before Dec 31, 2014 were included in the search.

### Inclusion and exclusion criteria

The inclusion criteria were: (i) cross-sectional community-based surveys, (ii) a definition of diarrhea of three or more loose stools in 24 h, (iii) a clear 2-week recall period, and (iv) published in Chinese or English. We excluded studies without the denominator (i.e., the total population of the catchment area), studies of specific diarrheal diseases (e.g., dysentery, rotavirus diarrhea), those of community intervention programs. We also excluded studies from the National Notifiable Disease Reported System of China because there is a high proportion of missing reports in the passive surveillance system, which leads to an underestimation of the ADI incidence [11].

### Data extraction

We removed the duplicates using EndNoteX7 software. Two researchers independently reviewed all abstracts to identify articles that assessed the incidence of ADI in sporadic diarrhea cases in the community. We reviewed each article and applied exclusion and inclusion criteria. If the same authors presented the data again in another study, only one study was included.

Data were independently extracted by two researchers and input to a predefined Microsoft Office Excel database, including name of the first author, year of publication, year(s) the study was conducted, case definition, location of the survey, sampling method, total sample size, total number of cases, and data for different subgroups. Disagreements were resolved by consulting the third researcher.

### Risk of bias

The Agency for Healthcare Research and Quality (AHRQ) recommends 11 items for assessing the quality of a cross-sectional/prevalence study [19, 20]. To date, this is the only accepted quality assessment tool for a cross-sectional study [21]. Additional file 1: Table S3 presents the AHRQ Cross-Sectional/Prevalence Study Quality Checklist. We did not conduct any sensitivity analysis of the quality assessments of the included studies, because there was no way to score the quality of the included studies based on the above assessment instrument. Two reviewers evaluated the quality of the enrolled studies independently.

### Data analysis

The data were entered into a database and analyzed by R-2.15.1 statistical software. The rate was logit transformed to generate pooled estimates [22]. The Q statistic (variation in effect size attributable to heterogeneity) was calculated to assess heterogeneity across studies. Studies were considered homogeneous when *p* > 0.10, in which case a fixed-effect model was used to generate pooled estimates; otherwise, the random-effect model was used [23]. When there was heterogeneity, subgroup analysis was performed to explore the source of heterogeneity. The Begg rank correlation test was used to assess publication bias. The 2-week prevalence of ADI was calculated by dividing the number of respondents reporting ADI in the 2 weeks prior to the interview by the total number of respondents. The incidence per person-year was calculated by multiplying the 2-week prevalence rate by 26, as the number of 2-week periods in a year is 26.07 (365/14).

## Results

**Fig. 1:**
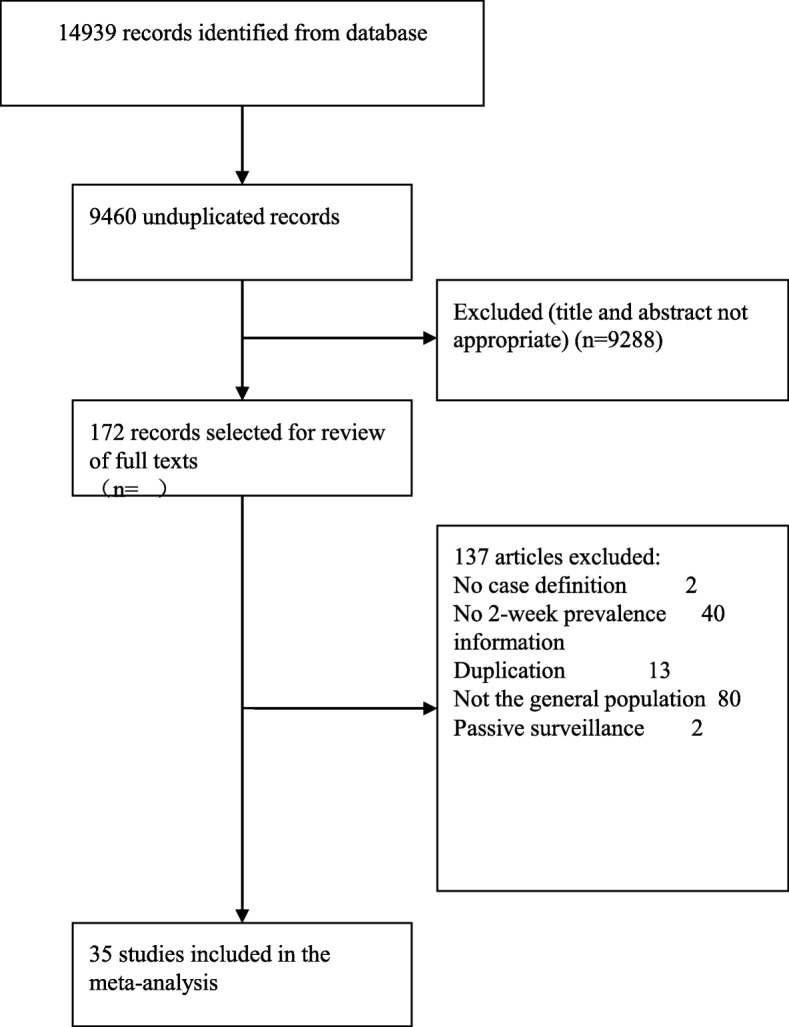
Flow of information through the systematic review

A total of 9460 unduplicated studies were identified (Fig. 1). After screening titles and abstracts, 172 reports were selected for full-text assessment. Finally, 35 studies were used for the meta-analysis (Additional file 2: Table S2).

### Characteristics of included studies

The included 35 studies were all performed in China and covered 16 provinces; 19 studies were conducted in east China, 10 in west China, three in central China, and two-thirds in south China. Furthermore, 17 studies were conducted in rural areas, four in urban areas, and 14 included both rural and urban regions. The surveys were conducted from 1984 to 2012, and more than half were conducted after 2000. The most frequent method used was face-to-face interviews, and only one telephone survey study was conducted. Table 1 and Additional file 3: Table S1 summarize the main and detailed characteristics, respectively, of the 35 studies included in this study.

**Table 1:** Main characteristics of studies included in the meta-analysis (*N* = 35)

Variable	No. of studies (n)	Proportion (% = n/N)
Time period^a^		
1980–1989	11	31
1990–1999	3	8
2000–2012	19	53
Unknown	3	8
Study area		0
Rural	17	49
Urban	4	11
Rural+urban (combined)	14	40
East	19	54
Central	3	9
West	10	29
Multi-province	2	6
Unknown	1	3
North	8	23
South	24	69
Multi-province	2	6
Unknown	1	3
Survey mode		0
Face-to-face survey	34	97
Telephone survey	1	3
Season		0
Spring	1	3
Summer	16	46
Autumn	1	3
Winter	2	6
Multi-season	14	40
Unknown	1	3

^a^one study was conducted from 1986 to 1996, and we divided it into 1980–1989 and 1990–1999. Spring: March, April, and May; summer: June, July, and August; autumn: September, October, and November; winter: December, January, and February

### Incidence of ADI in the community in China

**Fig. 2:**
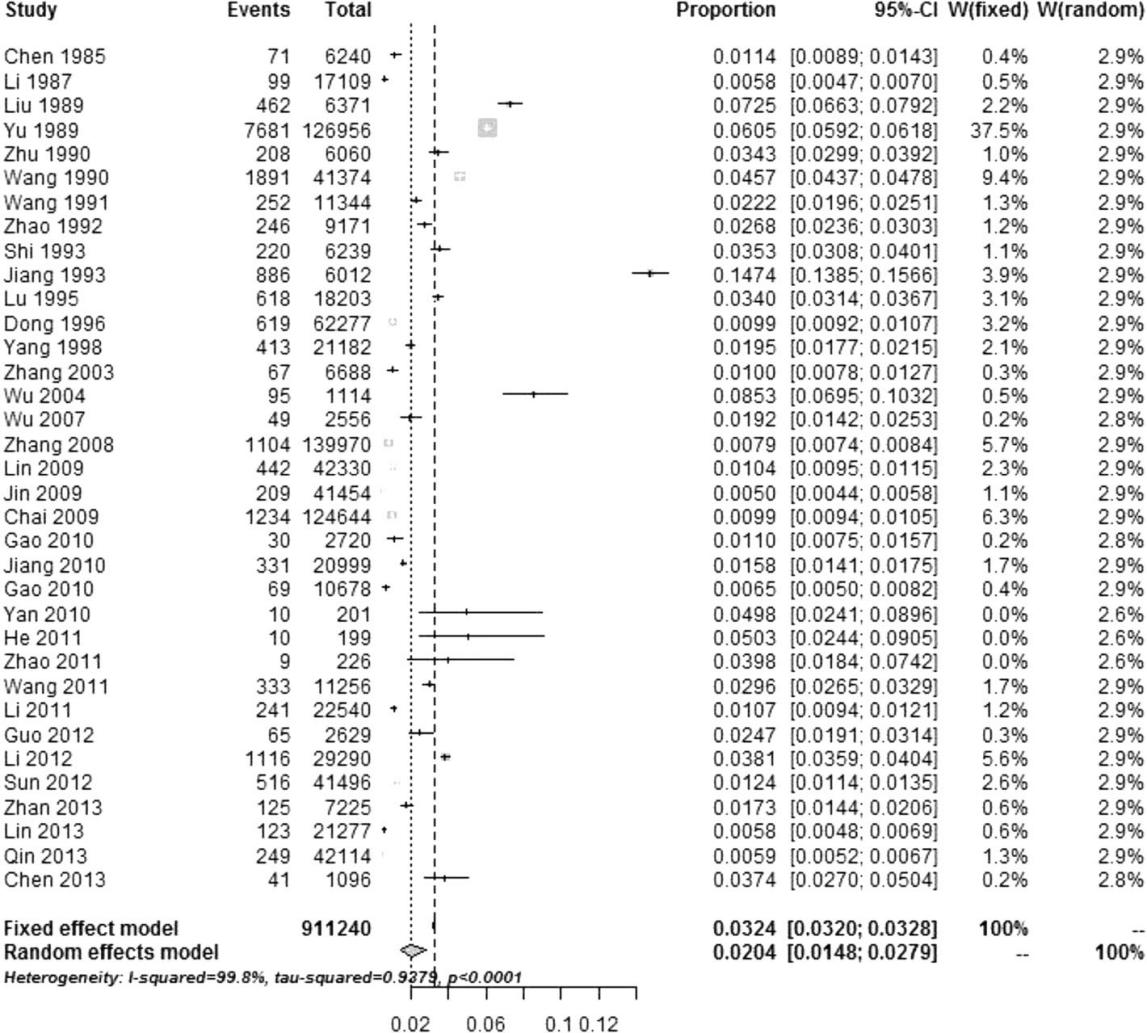
Forest plot of studies in the meta-analysis

The pooled estimate of 2-week prevalence of ADI is presented in Table 2 and Fig. 2. The 2-week prevalence of ADI in the pooled sample of 911,240 individuals was 2.04% (95%CI: 1.48–2.79), and the corresponding annual incidence of ADI was estimated as (365/14)×(2.04/100) = 0.53 (95% CI: 0.38–0.73) episodes per person-year.

**Table 2:** Overall and subgroup incidences of ADI

Variable	Cases/Total (No. of studies)	Pooled prevalence prior to 2-week period (%)	95%CI of prevalence (%)	Annual incidence per person-year^a^	95%CI of incidence per person-year	Q-value (*p*-value)
Overall	20,134/911240 (*n* = 35)	2.04	1.48–2.79	0.53	0.38–0.73	15,062.69 (0.000)
Gender						
Male	3428/196522 (*n* = 15)	2.22	1.30–3.77	0.58	0.34–0.98	3382.42 (0.000)
Female	3234/202362 (*n* = 15)	2.00	1.18–3.37	0.52	0.31–0.88	3074.44 (0.000)
Age group (year)						
0–4	3832/43463 (16)	5.51	3.76–8.01	1.43	0.98–2.08	1562.38 (< 0.0001)
5–14	3095/103720 (16)	2.10	1.38–3.18	0.55	0.36–0.83	1570.99 (< 0.0001)
≥15	10,325/496007 (16)	2.01	1.28–3.14	0.52	0.33–0.82	1808.43 (< 0.0001)
Time period						
1980–1989	12,142/243664 (*n* = 11)	3.17	2.23–4.49	0.82	0.60–1.27	2378.81 (0.000)
1990–1999	1524/94874 (*n* = 3)	1.89	0.86–4.13	0.49	0.22–1.07	476.52 (0.000)
2000–2012	6066/506591 (*n* = 19)	1.84	1.34–2.53	0.48	0.35–0.66	2596.51 (0.000)
Study area						
Urban	2510/267115 (*n* = 14)	1.28	0.80–2.06	0.33	0.21–0.54	1740.58 (0.000)
Rural	9445/486495 (*n* = 27)	1.94	1.38–2.72	0.50	0.36–0.71	6937.73 (0.000)
East	4800/415033 (*n* = 20)	1.48	1.11–1.97	0.38	0.29–0.51	1761.12 (0.000)
Central	2704/109382 (*n* = 4)	1.85	0.75–4.46	0.48	0.20–1.16	1054.45 (0.000)
West	4616/248613 (*n* = 11)	2.64	1.32–5.22	0.69	0.34–1.36	5396.89 (0.000)
South	7107/580306 (*n* = 25)	1.61	1.18–2.21	0.42	0.47–0.55	4179.49 (0.000)
North	5013/192722 (*n* = 10)	2.52	1.37–4.58	0.66	0.36–1.19	3737.22 (0.000)
Season						
Spring	1885/135064 (*n* = 9)	0.82	0.41–1.63	0.20	0.11–0.42	1275.56 (0.000)
Summer	15,131/440994 (*n* = 26)	2.33	1.71–3.16	0.61	0.44–0.82	6823.59 (0.000)
Autumn	1214/113349 (*n* = 9)	1.02	0.70–1.49	0.27	0.18–0.39	329.64 (0.000)
Winter	1080/132643 (*n* = 12)	0.94	0.77–1.15	0.24	0.20–0.30	95.50 (0.000)

All studies in the tables used the random-effect model (*p* < 0.1)

*CI* confidence interval, *Q-value* the value of the test for subgroup differences

^a^the incidence per person-year was calculated by multiplying the 2-week prevalence rate by 26, as there are 2607(365/14) 2-week periods in a year

### Demographic distribution

Sixteen studies included 196,522 males and 202,362 females. There was a male:female ratio of 51.5:48.5 in cases of ADI, and we found a statistically significant difference in the 2-week prevalence rates of ADI between males and females (2.22% vs 2.00%; OR = 1.11, 95% CI: 1.05–1.16).

Age was also significantly associated with ADI. The 2-week prevalence of ADI was highest in the < 5 YOA group, and the pooled estimate was 5.51%. The 2-week prevalence of ADI in those 5–14 years and≥15 years were quite similar (2.10 and 2.01%, respectively). The annual incidence of ADI among children < 5 YOA was 2.6- and 2.8-fold greater than those among 5–14 YOA and ≥15 YOA, respectively.

### Geographical distribution

Study sites were considered to be rural or urban according to the lifestyle of the residents. The pooled 2-week prevalence of ADI in rural areas was 1.5-fold (1.94, 95% CI: 1.38–2.72) higher than that in urban areas (1.28, 95% CI: 0.8–2.06).

We also classified studies by location according to the defined climate difference line of China: north of the Qinling Mountains and the Huaihe River were considered north China, and others were considered south China. The pooled estimate of the 2-week prevalence of ADI in north China (2.52, 95% CI: 1.37–4.58) was higher than that in south China (1.61, 95% CI: 1.18–2.21).

Studies were divided into different sub-groups according to economic level (from high to low): east China, central China, and west China [24]. The 2-week prevalence of ADI in east, central, and west China were 1.48% (95% CI: 1.11–1.97), 1.85% (95% CI: 0.75–4.46), and 2.64% (95% CI: 1.32–5.22), respectively. It was apparent that ADI was most prevalent in the western region.

### Trend by year

To determine the prevalence of ADI over time, we divided these studies into three time frames according to survey date. The random-effect model pooled estimate of the 2-week prevalence of ADI was 3.17% (95% CI: 2.23–4.49) before 1990, 1.89% (95% CI: 0.86–4.13) during 1990–1999, and 1.84% (95% CI: 1.34–2.53) after 2000. Apparently, the prevalence of ADI decreased slowly over the last three decades, although the rates of ADI were remarkably consistent for the 20 years after 1990.

### Seasonal distribution

The studies were also divided according whether they were conducted in the spring, summer, autumn, and winter to examine ADI epidemiology by season. The prevalence of ADI was the highest during summer (2.33, 95% CI: 1.71–3.16), followed by autumn (1.02, 95% CI: 0.70%–1.49), winter (0.94, 95% CI: 0.77–1.15), and spring (0.82, 95% CI: 0.41–1.63).

### Risk of bias

All 35 studies were cross-sectional in design. Thus, the AHRQ Cross-Sectional/ Prevalence Study Quality Checklist was applied to assess the risk of bias (Additional file 1: Table S3 and Additional file 4: Table S4). It should be noted that many studies were found to have a high risk of bias in items 7, 9, and 10, which indicated that the research did not adequately report the methods used by the primary studies for handling the missing data and calculating the response rates, which may influence confidence in the results. Furthermore, the Begg rank correlation test indicated no evidence of publication bias for reports of the 2-week prevalence of ADI (Z = 0.838, *p* = 0.402).

## Discussion

The current meta-analysis demonstrated that ADI remains an important public health problem causing a relatively large burden of illness in communities in China. Based on the current estimate of 0.53 ADI episodes per person-year, 725 million episodes of ADI occurred each year throughout China in 2014 [25]. The pooled estimate of the rate of ADI (0.53) is comparable to that of a similar retrospective study with a 28-day recall period conducted in China. Although the case definitions and recall period were different, that study reported 0.56 episodes of acute gastrointestinal illness (AGI) each year [15]. In Ireland, the United States, Indonesia, Australia, and Canada, retrospective studies using similar case definitions reported 0.44, 0.6, 0.65, 0.83, and 0.99 episodes of ADI per person per year, respectively [8, 14, 26]. Our estimate of ADI incidence in China was higher than that in Ireland but lower than that in the majority of developed countries. The observed differences between countries could be related to different exposure levels based on lifestyle differences, such as food consumption habits [15]. Although these studies were conducted a few years ago, a study conducted in the United States found no apparent decline in ADI over the last decade [8]. The burden of ADI remains significant worldwide [1].

Our results show that the risk for ADI is higher for children < 5 YOA compared with that for older individuals, which is consistent with other population-based studies [8, 13–15, 26]. This is not unexpected, as ADI comprises one of the leading causes of morbidity and mortality for those < 5 YOA worldwide [3]. This may be due to the relatively underdeveloped immune systems, the small infectious dose required, the close contact with other children in nurseries, and the contact with pets among this population [13]. Contrary to most other studies [8, 14, 27], but similar to two studies in Malta and Indonesia [13, 26], this study found a slightly higher rate of ADI in males than in females. This may be because males have a greater chance of wandering into unsanitary surroundings and being exposed to more enteric pathogens in the outdoors compared with females [28]. Alternatively, another study reported that the different incidence rates of ADI between males and females may also be related to the recall period and the season in which the study was conducted [29]. More studies are needed to clarify this issue.

In the current study, the rate of ADI was higher in rural regions than in urban regions, and east China showed the lowest rate of ADI compared with central China and west China. In fact, diarrheal epidemiology varied greatly by geographic regions, which may be related primarily to sanitation, drinking water quality, personal hygiene, education level, and economic development [30]. In general, the public health conditions, living environments, and sewage systems in urban and/or upper-class economic areas (east China) are relatively better than those in most rural and/or poor areas (central and west China); thus, the people living in the latter are more likely to be affected by enteric pathogens or be influenced by other risk factors [30]. This indicates that the improvement of environmental sanitation is the most important issue involved in the prevention and control of ADI in the rural and/or poor areas.

We found a significant decrease in the reported prevalence of ADI over time, from 0.82 to 0.48 per person per year, which was attributed to the widespread adoption of public health measures during those years. To reduce the burden of ADI, the Chinese government took many measures, such as reconstructing water supplies and lavatories in rural and poor areas, health education, greater encouragement of breast-feeding, and so on [31, 32]. However, we also observed that the rate of ADI declined slightly over the last two decades after 1990, even though the aforementioned measures were initiated prior to that time. This finding is consistent with a previous report in the United States that showed remarkably consistent rates of ADI over time [8]. The stable rates of ADI over the last two decades in China indicates the importance of improving our understanding of the specific causes of and effective preventive methods for ADI through additional research.

In term of the causes of ADI, foodborne illnesses have a primary role according to the WHO report [33]. In the United States, 25–30% of ADI cases are thought to be caused by foodborne illnesses [34], and the corresponding proportion was 32% in Australia [35]. In China, 36.5–55.5% of ADI cases reported that consumption of contaminated food was the cause of their illness, although people seldom actually know the cause of their ADI [15, 18]. Additionally, the observed peak of incidence during the summer months, which is consistent with previous surveys of AGI [15, 18], has been associated with bacterial foodborne illnesses [36, 37]. Indeed, as it is almost impossible for a retrospective cross-sectional study to collect stool samples and suspected food in cases of ADI, it is very difficult to identify the actual cause and etiology of this condition. Moreover, antibiotic abuse and the fact that not all cases of ADI present to healthcare facilities greatly affect the results of hospital-based or laboratory-based surveillance [15]. When conditions permit, a prospective cohort study may be a better way to determine the actual incidence rates, causes, and pathogen-specific burden of ADI. Identifying the etiologies of ADI and the proportion of ADI cases attributable to foodborne and other sources will be crucial to the implementation of effective prevention measures.

Our study has several limitations. The recall period and case definition are two key factors that may affect the final results in a retrospective cross-sectional study of ADI [29, 38, 39]. A recall bias may arise because there are likely to be differences in the ability to remember experiences according to context [13]. One form of recall bias, called ‘telescoping’ (i.e., the tendency for people to displace events in time), is especially important in this type of study; this bias would tend to yield an overestimate of the frequency of ADI [40]. To reduce this bias, only cross-sectional studies with a 2-week recall period were included in the current analysis. Furthermore, consistent with WHO guidelines [41], diarrhea was defined as three or more loose stools in 24 h. Only studies meeting the above definition were included in our analysis. Nonetheless, the primary limitation of this meta-analysis relates to the heterogeneity in ADI cross-sectional surveys.

## Conclusions

To the best of our knowledge, the current study provides the first population-based estimate of the magnitude and distribution of ADI in the general Chinese population using a meta-analytic method. ADI remains a significant health burden in the community, especially among those < 5 YOA and in rural/poor regions in China. Overall, the temporal distribution peaked in summer months. However, the rates of ADI were remarkably consistent throughout the two decades since 1990, which placed a substantial burden on the healthcare system. Further research, including a prospective cohort study of the pathogen-specific burden of ADI, is necessary to better estimate the burden of ADI and develop effective prevention measures.

## Additional files

Additional file 1: **Table S3.** ARHQ Methodology Checklist for Cross-Sectional/Prevalence Study. (DOCX 13 kb)

Additional file 2: **Table S2.** References included in the final analysis. (DOCX 17 kb)

Additional file 3: **Table S1.** Detailed characteristics of studies included in the meta-analysis. (DOCX 18 kb)

Additional file 4: **Table S4.** Quality evaluation of the 35 studies included in the meta-analysis. (DOCX 14 kb)

## Abbreviations

ADI: Acute diarrheal illness
AGI: Acute gastrointestinal illness
CI: Confidence interval
OR: Odds ratio
WHO: World Health Organization
YOA: Years of age

## References

[1] Mathers C, Lopez AD, Murray C, Stein C (2001). The global burden of disease 2000 project: aims, methods and data sources.

[2] Guerrant RL, Kosek M, Moore S, Lorntz B, Brantley R, Lima AA (2002). Magnitude and impact of diarrheal diseases. Arch Med Res.

[3] Liu L, Oza S, Hogan D, Perin J, Rudan I, Lawn JE, Cousens S, Mathers C, Black RE (2015). Global, regional, and national causes of child mortality in 2000-13, with projections to inform post-2015 priorities: an updated systematic analysis. Lancet (London, England).

[4] Fischer Walker CL, Perin J, Aryee MJ, Boschi-Pinto C, Black RE (2012). Diarrhea incidence in low- and middle-income countries in 1990 and 2010: a systematic review. BMC Public Health.

[5] Walker CL, Black RE (2010). Diarrhoea morbidity and mortality in older children, adolescents, and adults. Epidemiol Infect.

[6] van den Brandhof WE, De Wit GA, de Wit MA, van Duynhoven YT (2004). Costs of gastroenteritis in the Netherlands. Epidemiol Infect.

[7] Reid R, Scallan S, Bruce D (2011). Cost and effectiveness of protected learning time in primary care organisations. Educ Prim Care.

[8] Jones TF, McMillian MB, Scallan E, Frenzen PD, Cronquist AB, Thomas S, Angulo FJ (2007). A population-based estimate of the substantial burden of diarrhoeal disease in the United States; FoodNet, 1996-2003. Epidemiol Infect.

[9] Chen Y, Guo Y, Wang Z, Liu X, Liu H, Dai Y, Tang Z, Wen J (2010). Foodborne disease outbreaks in 2006 report of the National Foodborne Disease Surveillance Network, China. J Hyg Res.

[10] Ran L, Wu S, Gao Y, Zhang X, Feng Z, Wang Z, Kan B, Klena JD, Lo Fo Wong DM, Angulo FJ (2011). Laboratory-based surveillance of nontyphoidal Salmonella infections in China. Foodborne Pathog Dis.

[11] Qin SW, Lü HK, Yu Z, Chen EF, Zhang J. A comparison of incidences of diarrhea between a population-based study and the disease surveillance system in Zhejiang province. Chin Rural Health Serv Adm 2013;33:1373–1377.

[12] Mao XD (2010). Study on epidemiological characteristic and disease burden of bacterial foodborne disease in China, during 2003-2008.

[13] Gauci C, Gilles H, O’Brien S, Mamo J, Stabile I, Ruggeri FM, Gatt A, Calleja N, Spiteri G (2007). The magnitude and distribution of infectious intestinal disease in Malta: a population-based study. Epidemiol Infect.

[14] Scallan E, Majowicz SE, Hall G, Banerjee A, Bowman CL, Daly L, Jones T, Kirk MD, Fitzgerald M, Angulo FJ (2005). Prevalence of diarrhoea in the community in Australia, Canada, Ireland, and the United States. Int J Epidemiol.

[15] Chen Y, Yan WX, Zhou YJ, Zhen SQ, Zhang RH, Chen J, Liu ZH, Cheng HY, Liu H, Duan SG (2013). Burden of self-reported acute gastrointestinal illness in China: a population-based survey. BMC Public Health.

[16] Zhang J, Liu M (2008). Current situation on the treatment modules of diarrhea cases in 12 counties/cities of Guangdong, Henan and Gansu provinces in China. Zhonghua Liu Xing Bing Xue Za Zhi.

[17] Zhang YL, Ma J, Zhang YW, Hao ZY, Wang YY (2006). Analysis on incidence of shigellosis in rural area of Zhengding county in Hebei province. Chin J Public Health.

[18] Sang X-L, Liang X-C, Chen Y, Li J-D, Li J-G, Bai L, Sun J-Y (2014). Estimating the burden of acute gastrointestinal illness in the community in Gansu Province, northwest China, 2012–2013. BMC Public Health.

[19] Rostom A, Dubé C, Cranney A. Celiac Disease. Rockville (MD): Agency for Healthcare Research and Quality (US). https://www.ncbi.nlm.nih.gov/books/NBK35156/. Accessed Sept 2004.

[20] Zeng X, Zhang Y, Kwong JS, Zhang C, Li S, Sun F, Niu Y, Du L (2015). The methodological quality assessment tools for preclinical and clinical studies, systematic review and meta-analysis, and clinical practice guideline: a systematic review. J Evid Based Med.

[21] Sanderson S, Tatt ID, Higgins JP (2007). Tools for assessing quality and susceptibility to bias in observational studies in epidemiology: a systematic review and annotated bibliography. Int J Epidemiol.

[22] Luo ML, Tan HZ, Zhou Q, Wang SY, Cai C (2013). Realizing the meta-analysis of single rate in R software. J Evid Based Med.

[23] Higgins JP, Thompson SG, Deeks JJ, Altman DG (2003). Measuring inconsistency in meta-analyses. BMJ (Clin Res Ed).

[24] Yuan J (2006). The economic regionalization research in China. Commercial Times.

[25] National Bureau of Statistics of the People’s Republic of China (2015). China statistical yearbook.

[26] Simanjuntak CH, Punjabi NH, Wangsasaputra F, Nurdin D, Pulungsih SP, Rofiq A, Santoso H, Pujarwoto H, Sjahrurachman A, Sudarmono P (2004). Diarrhoea episodes and treatment-seeking behaviour in a slum area of North Jakarta, Indonesia. J Health Popul Nutr.

[27] Kuusi M, Aavitsland P, Gondrosen B, Kapperud G (2003). Incidence of gastroenteritis in Norway--a population-based survey. Epidemiol Infect.

[28] Chowdhury F, Khan IA, Patel S, Siddiq AU, Saha NC, Khan AI, Saha A, Cravioto A, Clemens J, Qadri F (2015). Diarrheal illness and healthcare seeking behavior among a population at high risk for diarrhea in Dhaka, Bangladesh. PLoS One.

[29] Thomas MK, Perez E, Majowicz SE, Reid-Smith R, Albil S, Monteverde M, McEwen SA (2010). Burden of acute gastrointestinal illness in Galvez, Argentina, 2007. J Health Popul Nutr.

[30] Yang JJ (2008). Research on diarrhea diseases: prevalent features and economic burden of Gansu Province.

[31] Xiao DL, Song YT, Wang CA, Cai RH, Nan JH, et al. The current situation of diarrheal disease control in China. Chin J Epidemiol 1996;5:296–298.

[32] Jin T, Tao Y (2005). The position and role of disease prevention and control institutions in safe drinking water and sanitary washroom work in rural areas. Chin Health Serv Manag.

[33] World Health Organization (2015). WHO estimates of the global burden of foodborne diseases.

[34] McCabe-Sellers BJ, Beattie SE (2004). Food safety: emerging trends in foodborne illness surveillance and prevention. J Am Diet Assoc.

[35] Hall G, Kirk MD, Becker N, Gregory JE, Unicomb L, Millard G, Stafford R, Lalor K (2005). Estimating foodborne gastroenteritis, Australia. Emerg Infect Dis.

[36] Patz JA, Engelberg D, Last J (2000). The effects of changing weather on public health. Annu Rev Public Health.

[37] Kovats RS, Edwards SJ, Charron D, Cowden J, D’Souza RM, Ebi KL, Gauci C, Gerner-Smidt P, Hajat S, Hales S (2005). Climate variability and campylobacter infection: an international study. Int J Biometeorol.

[38] Majowicz SE, Hall G, Scallan E, Adak GK, Gauci C, Jones TF, O’Brien S, Henao O, Sockett PN (2008). A common, symptom-based case definition for gastroenteritis. Epidemiol Infect.

[39] Cantwell LB, Henao OL, Hoekstra RM, Scallan E (2010). The effect of different recall periods on estimates of acute gastroenteritis in the United States, FoodNet population survey 2006-2007. Foodborne Pathog Dis.

[40] van den Brink M, Bandell-Hoekstra EN, Abu-Saad HH (2001). The occurrence of recall bias in pediatric headache: a comparison of questionnaire and diary data. Headache.

[41] World Health Organization (2005). The treatment of diarrhoea: a manual for physicians and other senior health workers.

