# Supplementary material for: Incidence of acute diarrheal illness in Chinese communities: a meta-analysis
Source: BMC Gastroenterol. 2018 Jul 13;18:114. doi: 10.1186/s12876-018-0839-2 (PMC6045875; doi:10.1186/s12876-018-0839-2)
Supplement: Supplementary file 1 — Table S3. ARHQ Methodology Checklist for Cross-Sectional/Prevalence Study. (DOCX 13 kb) [file 12876_2018_839_MOESM1_ESM.docx]

Table S3. ARHQ Methodology Checklist for Cross-Sectional/Prevalence Study

Website: http://www.ncbi.nlm.nih.gov/books/NBK35156/

| Item | Yes | No | Unclear |
| --- | --- | --- | --- |
| ① Define the source of information (survey, record, review) |  |  |  |
| ②List inclusion and exclusion criteria for exposed and unexposed subjects (cases and controls) or refer to previous publications |  |  |  |
| ③ Indicate time period used for identifying patients |  |  |  |
| ④Indicate whether subjects were consecutive if not population-based |  |  |  |
| ⑤ Indicate if evaluators of subjective components of study were blind to other aspects of the status of the participants |  |  |  |
| ⑥ Describe any assessments undertaken for quality assurance purposes (e.g., test/retest of primary outcome measurements) |  |  |  |
| ⑦ Explain any exclusions of data from analysis |  |  |  |
| ⑧ Describe how confounding was assessed and/or controlled |  |  |  |
| ⑨ If applicable, explain how missing data were handled in the analysis |  |  |  |
| ⑩ Summarize patient response rates and completeness of data collection |  |  |  |
| ⑪ Clarify what follow up, if any, was expected and the percentage of patients for which incomplete data or follow-up was obtained |  |  |  |
