# Supplementary material for: Incidence of acute diarrheal illness in Chinese communities: a meta-analysis
Source: BMC Gastroenterol. 2018 Jul 13;18:114. doi: 10.1186/s12876-018-0839-2 (PMC6045875; doi:10.1186/s12876-018-0839-2)
Supplement: Supplementary file 2 — Table S2. References included in the final analysis. (DOCX 17 kb) [file 12876_2018_839_MOESM2_ESM.docx]

Table S2. References included in the final analysis

| Code | Reference information (All in Chinese) |
| --- | --- |
| 1 | Chen LF, Ren YH, Pan ZM: **The incidence and mortality rates of diarrhea disease survey in Guangzhou city**. *Guangzhou Medical Journal* 1985, 4:2–3. |
| 2 | Li JJ, Lin P, Li GS, Shi XC, Liu LC, Huang CM, Liu SP, Zhong ZH: **The incidence and mortality rates of diarrhea disease survey in Shantou city.** *Guangdong Wei Sheng Fang Yi* 1987, (4):6–8. |
| 3 | Liu B, Lai CL, Tang ZZ, Dong BQ, Shen G, Tang ZM, Zou GY, Zhao BY, Li HL, Liang ZY, Fang SY: **The epidemiology of diarrhea disease in Yishan county, Guangxi province**. *Guangxi Medical Journal* 1989, 11(2):87–89. |
| 4 | Yu WL: **Household survey of diarrhoeal disease control in P. R. China**. *Chin J Epidemiol*1989, 10(5):257–260. |
| 5 | Zhu YH, Wang HL, Mou HY, Xing YG, Niu JS, Nan RX, Wu Q: **Household survey of diarrhoeal disease control in Fengzhen county**. *Inner Mongolia Medical Journal* 1990, 10(4):39–40. |
| 6 | Wang Z: **Household survey of infectious intestinal disease control in rural regions in Henan province**. *Henan Journal of Preventive Medicine* 1990, 1(2):128–131. |
| 7 | Wang ZQ, Ouyang B, Luo XQ, Luo SH: **The report of diarrhoeal disease control in Emeishan city, Sichuan province**. *Journal of Preventive Medicine Information* 1991, 37(3):169–170. |
| 8 | Zhao J, Zhang LL: **Household survey of infectious diarrheal disease control in Fentai district, Beijing city**. *Chin J Public Health* 1992, 8(10):434. |
| 9 | Shi XC, Lin P, Li GS, Chen TY, Liu LC: **Household survey of diarrhoeal disease control in Puning country, Guangdong province**. *Guangdong Medical Journal* 1993, 14(1):20–22. |
| 10 | Jiang ZK, Li SZ: **Household survey of diarrhoeal disease control in Tibet**. T*ibetan Medicine* 1993, (2):64–65. |
| 11 | Lu GZ, Xu S, Dai GS, Shi ZB, Lu Z, Zhang SH: **Household survey of infectious diarrhoeal disease control in Linyi city, Shandong province**. *Journal of Linyi Medical College* 1995, 17(3):253–256. |
| 12 | Dong BQ, Liu FY, Chen FQ, Gan ZG, Li JD, Yi YF, Li YS, Ya CM: **Household survey of diarrhoeal disease in 5 counties (or cities) in Guangxi province**. *Applied Prev Med* 1996, 2(4):205–208. |
| 13 | Yang J, Sun DQ, Chen ZA, Zhang SX: **Investigation and study of the infectious diarrhoea in rural area crowd of Rudong county from 1986**–**1996.** *Modern Prevention Medicine* 1998, 25(2):164–166. [Please check this translation.] |
| 14 | Zhang YX, Yang SH, Cheng YC, Cai LB: **Investigation and study of the missing reports of diarrhoeal disease in Yandu county from 2000**–**2001**. *Modern Preventive Medicine* 2003, 30(2):243–245. |
| 15 | Wu XY, Zeng Q: **Prevalence and impact of diarrhea in Chongqing urban area.**  *Journal of Chongqing Medical University* 2004, 29(2):216–218. |
| 16 | Zhao ZQ: **The analysis of the diarrhea disease survey in Meihekou city**. *Chinese Community Doctors* 2007, 9(23):245. |
| 17 | Zhang J, Liu M: **Current situation on the treatment modules of diarrhea cases in 12 counties/cities of Guangdong, Henan and Gansu provinces in China**. *Chin J Epidemiol* 2008, 29(10): 989–993. |
| 18 | Lin M, Dong BQ, Liang DB, Li YH, Wu XH, Yin K, Meng ZH, Qin WW, Zhong LQ, Pan MF: **Study on prevalence and disease burden of infectious diarrhea in Guangxi**. *Chin J Public Health* 2009, 25(3):246–348. |
| 19 | Jin LJ, Yuan H, Zhang H, Leng J, Xin ZL, Su L, Hao CX, Tang XY: **Current condition investigation of diarrhea in Sichuan province.** *J Prev Med Inf* 2009, 25(3):183–186. |
| 20 | Chai CL, Lu HK, Yu Z, Qin SW, Wang XX, Chen K, Xie SY: **Community-based study on disease burden of diarrhea in Zhejiang province**. *Chin J Epidemiol* 2009, 30(10):1005–1009. |
| 21 | Gao L, Ye Z, Yang CJ，Xu JR: **Survey of two-week prevalence of diarrhea and its influential factors in rural inhabitants of Xiangshan island**. *China Tropical Medicine* 2010, 10(6):689. |
| 22 | Jiang LF, Wang FJ, Wang YL, Guo JF: **Incidence of diarrhea and hospital visits**  **of patients with diarrhea in Xiaoshan district, Hangzhou city**. *Chin J Nat Med* 2010, 12(3):198–200. |
| 23 | Gao L, Yang CJ, Qin SW, Gao YJ, Lu HK: **Current situation on the treatment modes of diarrhea cases in rural area**. *Inter J Epidemiol Infect Dis* 2010, 37(2):99–102. |
| 24 | Yan SY, Liu H, Chen YQ, Zheng B, Xu HZ, Gao WW, Wang LW: **Occurrence of food-borne diarrhea in Jing’an district of Shanghai and analysis on countermeasures**. *Chinese Journal of Food Hygiene* 2010, 22(3):277–280. |
| 25 | He AN, Ma LF, Xu J, Zhou WM: **Household survey of food-borne diarrhea in Huangpu district, Shanghai city**. *Chinese Primary Health Care* 2011, 25(8):90–91. |
| 26 | Zhao LF, Liu LJ, Tan YY: **Household survey of food-borne diarrhea in Minhang district, Shanghai city.** *Chinese Primary Health Care* 2011, 25(1):68-69. |
| 27 | Wang S. **Study on burden of diarrheal diseases and cost-benefit analysis due to water supply and sanitation facilities in typical rural areas**. *Chinese Center for Disease Control and Prevention, Beijing*. 2011. |
| 28 | Li XH, Duan JJ, Chen YZ, Han TW: **Current situation on the epidemic characteristics of diarrheal disease in Zhengzhou city.** *Modern Preventive Medicine* 2011, 38(1):8–10. |
| 29 | Guo ZJ, Chen FG, Zhao W: **Analysis of influencing factors of environmental sanitation and behavior habits associated with diarrhea in rural areas of Shijiazhuang.** *Practical Preventive Medicine* 2012, 19(9):1288–1290. |
| 30 | Li XH, Wang Y, Liu Y, Zeng XM, Mu Y, Wen Y, Wang ZW, Li MC, Pan Q, Cao Y: **Diarrheal disease prevalence and its medical care status among residents in Chengdu**. *Journal of Occupational Health and Damage* 2012, 27(4):220–222. |
| 31 | Sun Z, Deng J, Xie L, Jia YJ, Wang FJ, Huang RJ, Kao QJ: **A survey of the incidence and status of health-seeking behavior of patients with diarrhea among urban and rural residents in Hangzhou city**. *Zhengjia Preventive Medicine* 2012, 24(3):4–6. |
| 32 | Zhan XH, Lin ZS, Wang K: **Analysis of influencing factors of environmental sanitation and behavior habits associated with diarrhea in rural areas of Fujian province**. *Chinese Primary Health Care* 2013, 27(7):103–104. |
| 33 | Lin Y, Wang JR, Fu XF, Wang XJ, Shen ZM, Jiang WP, Xu RQ, Shen GC: **The burden survey of diarrhea in the community in Jiaxing city.** *Chin J of PHM* 2013, 29(2):58–160. |
| 34 | Qin SW, Lu HK, Yu Z, Chen EF, Zhang J: **A comparison of incidences of diarrhea between a population-based study and the disease surveillance system in Zhejiang province.** *Chinese Rural Health Service Administration* 2013, 33(12):1373–1376. |
| 35 | Chen J, Li ZC, Gao XF, Du HL, Yu L, Ren HY, Zhu YX: **Toilet retrofit in rural areas of China: impact factors and effect analysis**. *Chinese Rural Health Service Administration* 2013, 33(2):181–183. |
