# Supplementary material for: Incidence of acute diarrheal illness in Chinese communities: a meta-analysis
Source: BMC Gastroenterol. 2018 Jul 13;18:114. doi: 10.1186/s12876-018-0839-2 (PMC6045875; doi:10.1186/s12876-018-0839-2)
Supplement: Supplementary file 3 — Table S1. Detailed characteristics of studies included in the meta-analysis. (DOCX 18 kb) [file 12876_2018_839_MOESM3_ESM.docx]

Table S1. Detailed characteristics of studies included in the meta-analysis

| Study | Province | Region | | | Survey date | Survey mode | Sampling  method | Sample size | Number of cases prior to 2-week period | Prevalence  (%) |
| --- | --- | --- | --- | --- | --- | --- | --- | --- | --- | --- |
| Chen LF 1985 | Guangdong | Rural/Urban | South | East | Jul 1984–Sep 1984 | Face to face | R,C | 6240 | 71 | 1.14 |
| Li JJ 1987 | Guangdong | Urban | South | East | Jun 1986–Mar 1987 | Face to face | S,R | 17109 | 99 | 0.58 |
| Liu B 1989 | Guangxi | Rural | South | West | Aug 1988 | Face to face | M,S,C,R | 6371 | 462 | 7.25 |
| Yu WL 1989 | Multi-province | Rural/Urban | Multi-province | Multi-province | Aug 1988 | Face to face | M,S,C,R | 126956 | 7681 | 6.05 |
| Zhu YH 1990 | Neimenggu | Rural | North | West | Aug 1988 | Face to face | M,S,R,C | 6060 | 208 | 3.43 |
| Wang Z 1990 | Henan | Rural | North | Central | Sep 1989 | Face to face | M,S,R,C | 41374 | 1891 | 4.57 |
| Wang ZQ 1991 | Sichuan | Rural | South | West | Aug 1989–Dec 1989 | Face to face | C | 11344 | 252 | 2.22 |
| Zhao J 1992 | Beijing | Rural/Urban | North | East | Oct 1988 | Face to face | NA | 9171 | 246 | 2.68 |
| Shi XC 1993 | Guangdong | Rural | South | East | Aug 1988 | Face to face | M,S,R,C | 6239 | 220 | 3.53 |
| Jiang ZK 1993 | Tibet | Rural | North | West | Sep 1988 | Face to face | M,S,R,C | 6012 | 886 | 14.74 |

Table S1. (continued)

| Study | Province | Region | | | Survey date | Survey mode | Sampling  method | Sample size | Number of cases prior to 2-week period | Prevalence  (%) |
| --- | --- | --- | --- | --- | --- | --- | --- | --- | --- | --- |
| Lu GZ 1995 | Shandong | Rural/Urban | North | East | Sep 1993 | Face to face | S,R | 18203 | 618 | 3.40 |
| Dong BQ 1996 | Guangxi | Rural | South | West | Jun 1995 | Face to face | C | 62277 | 619 | 0.99 |
| Yang J 1998 | Jiangsu | Rural | South | East | 1986–1996 | Face to face | M,S,C,R | 21182 | 413 | 1.95 |
| Zhang YX 2003 | Jiangsu | Rural | South | East | 2000–2001 | Face to face | C,R | 6688 | 67 | 1.00 |
| Wu XY 2004 | Chongqing | Urban | South | West | 2002 | Telephone survey | M,S,PPS,R | 1114 | 95 | 8.53 |
| Wu XH 2007 | Jilin | Rural | North | Central | Dec 2006–Mar 2007 | Face to face | C,R | 2556 | 49 | 1.92 |
| Zhang J 2008 | Guangdong/Henan/Gansu | Rural/Urban | Multi-province | Multi-province | Dec 2006–Sep 2007 | Face to face | M,S,C,R | 139970 | 1104 | 0.79 |
| Lin M 2009 | Guangxi | Rural/Urban | South | West | Aug 2007–Sep 2007 | Face to face | C,R | 42330 | 442 | 1.04 |

Table S1. (continued)

| Study | Province | Region | | | Survey date | Survey mode | Sampling  method | Sample size | Number of cases prior to 2-week period | Prevalence  (%) |
| --- | --- | --- | --- | --- | --- | --- | --- | --- | --- | --- |
| Jin LJ 2009 | Sichuan | Rural/Urban | South | West | Dec 2006–Sep 2007 | Face to face | M,S,C,R | 41454 | 209 | 0.50 |
| Chai CL 2009 | Zhejiang | Rural/Urban | South | East | Jul 2007–Apr 2008 | Face to face | M,S,C,R | 124644 | 1234 | 0.99 |
| Gao L 2010 | Zhejiang | Rural | South | East | NA | Face to face | M,C,R | 2720 | 30 | 1.10 |
| Jiang KF 2010 | Zhejiang | Rural | South | East | Jul 2007–Apr 2008 | Face to face | C,R | 20999 | 331 | 1.58 |
| Gao L 2010 | Zhejiang | Rural | South | East | Dec 2006–Sep 2007 | Face to face | M,S,C,R | 10678 | 69 | 0.65 |
| Yan SY 2010 | Shanghai | Urban | South | East | Dec 2008 | Face to face | C,R | 201 | 10 | 4.98 |
| He AN 2011 | Shanghai | Urban | South | East | Jul 2010 | Face to face | R | 199 | 10 | 5.03 |
| Zhao LF 2011 | Shanghai | Rural/Urban | South | East | Dec 2009 | Face to face | M,S,R | 226 | 9 | 3.98 |

Table S1. (continued)

| Study | Province | Region | | | Survey date | Survey mode | Sampling  method | Sample size | Number of cases prior to 2-week period | Prevalence  (%) |
| --- | --- | --- | --- | --- | --- | --- | --- | --- | --- | --- |
| Wang S 2011 | NA | Rural | NA | NA | Jul 2009–Sep 2009 | Face to face | M,S,R | 11256 | 333 | 2.96 |
| Li XH 2011 | Henan | Rural/Urban | North | Central | Dec 2006–Sep 2007 | Face to face | M,S,C,R | 22540 | 241 | 1.07 |
| Guo ZJ 2012 | Hebei | Rural | North | East | Aug 2011 | Face to face | S,R | 2629 | 65 | 2.47 |
| Li XH 2012 | Sichuan | Rural/Urban | South | West | Apr 2011–Jun 2011 | Face to face | C,R | 29290 | 1116 | 3.81 |
| Sun Z 2012 | Zhejiang | Rural/Urban | South | East | 2009 | Face to face | C,R | 41496 | 516 | 1.24 |
| Zhan XH 2013 | Fujian | Rural | South | East | Aug 2012–Sep 2012 | Face to face | S,R | 7225 | 125 | 1.73 |
| Lin X 2013 | Zhejiang | Rural/Urban | South | East | NA | Face to face | M,C,T,R | 21277 | 123 | 0.58 |
| Qin SW 2013 | Zhejiang | Rural/Urban | South | East | NA | Face to face | PPS,M,R,C,T | 42114 | 249 | 0.59 |
| Chen J 2013 | Sichuan | Rural | South | West | Jul–Aug, 2011 | Face to face | S,R | 1096 | 41 | 3.74 |

Note: NA, not available; R, random sampling; C, clustered sampling; S, stratified sampling; T, systematic sampling; M, multi-stage sampling; PPS, probability-proportionate-to-size sampling.
