# Supplementary material for: Incidence of acute diarrheal illness in Chinese communities: a meta-analysis
Source: BMC Gastroenterol. 2018 Jul 13;18:114. doi: 10.1186/s12876-018-0839-2 (PMC6045875; doi:10.1186/s12876-018-0839-2)
Supplement: Supplementary file 4 — Table S4. Quality evaluation of the 35 studies included in the meta-analysis. (DOCX 14 kb) [file 12876_2018_839_MOESM4_ESM.docx]

Table S4. Quality evaluation of the 35 studies included in the meta-analysis.

| Study code | ① | ② | ③ | ④ | ⑤ | ⑥ | ⑦ | ⑧ | ⑨ | ⑩ | ⑪ |
| --- | --- | --- | --- | --- | --- | --- | --- | --- | --- | --- | --- |
| 1 | Y | Y | Y | - | N | Y | N | Y | N | N | - |
| 2 | Y | Y | Y | - | N | Y | N | Y | N | N | - |
| 3 | Y | Y | Y | - | N | Y | N | Y | N | N | - |
| 4 | Y | Y | Y | - | N | Y | N | Y | N | N | - |
| 5 | Y | Y | Y | - | N | U | N | Y | N | N | - |
| 6 | Y | Y | Y | - | N | U | N | Y | N | N | - |
| 7 | Y | Y | Y | - | N | N | N | Y | N | N | - |
| 8 | Y | Y | Y | - | N | N | N | N | N | N | - |
| 9 | Y | Y | Y | - | N | N | N | N | N | N | - |
| 10 | Y | Y | Y | - | N | U | N | Y | N | N | - |
| 11 | Y | Y | Y | - | N | Y | N | N | N | N | - |
| 12 | Y | Y | Y | - | N | Y | N | Y | N | N | - |
| 13 | Y | Y | Y | - | N | Y | N | Y | N | N | - |
| 14 | Y | Y | Y | - | N | Y | N | Y | N | N | - |
| 15 | Y | Y | Y | - | N | Y | N | Y | N | Y | - |
| 16 | Y | Y | Y | - | N | U | N | Y | N | N | - |
| 17 | Y | Y | Y | - | N | Y | N | Y | N | N | - |
| 18 | Y | Y | Y | - | N | Y | N | Y | N | N | - |
| 19 | Y | Y | Y | - | N | Y | N | Y | Y | N | - |
| 20 | Y | Y | Y | - | N | N | N | Y | N | N | - |
| 21 | Y | Y | Y | - | N | Y | N | N | N | N | - |
| 22 | Y | Y | Y | - | N | Y | N | Y | Y | N | - |
| 23 | Y | Y | Y | - | N | Y | N | Y | Y | N | - |
| 24 | Y | Y | Y | - | N | Y | N | Y | N | N | - |
| 25 | Y | Y | Y | - | N | Y | N | Y | N | N | - |
| 26 | Y | Y | Y | - | N | Y | N | Y | N | N | - |
| 27 | Y | Y | Y | - | N | Y | N | Y | N | N | - |
| 28 | Y | Y | Y | - | N | Y | N | Y | Y | N | - |
| 29 | Y | Y | Y | - | N | Y | N | Y | N | N | - |
| 30 | Y | Y | Y | - | N | Y | N | N | Y | N | - |
| 31 | Y | Y | Y | - | N | Y | N | Y | Y | N | - |
| 32 | Y | Y | Y | - | N | Y | N | Y | N | N | - |
| 33 | Y | Y | Y | - | N | Y | N | N | N | N | - |
| 34 | Y | Y | Y | - | N | Y | N | Y | N | N | - |
| 35 | Y | Y | Y | - | N | Y | N | Y | N | Y | - |

Note: Y, yes; N, no; U, unclear. Items 4 and 11 were not appropriate for the studies included in the meta-analysis.
